# Effects of CrossFit™ versus regular training on physical fitness and skills in U12 basketball players: A randomized controlled trial

**DOI:** 10.1097/MD.0000000000043422

**Published:** 2025-07-25

**Authors:** Jian Gong, Lingqi Li, Quan Zhou, Jia Zhang

**Affiliations:** a Department of Physical Education, Graduate School, Pukyong National University, Busan, South Korea; b Guangzhou Social Welfare Institute, Guangzhou, Guangdong, China; c Huaihua Normal College, Huaihua City, China; d School of Physical Education, Chongqing University, Chongqing, China.

**Keywords:** adolescents, basketball training, CrossFit training, skill development, U12 basketball players

## Abstract

**Background:**

CrossFit™ (CF) training has gained popularity as a high-intensity functional training method, but its effectiveness in youth sports remains underexplored. This study examined the impact of CF on the physical fitness and basketball-specific skills of U12 athletes. The objective was to compare the effects of CF training versus regular training on physical performance and basketball-related abilities in U12 basketball players.

**Methods:**

A randomized controlled trial was conducted involving 40 male U12 basketball players. Participants were randomly assigned to either an experimental group (CF, n = 20) or a control group (regular training, n = 20) for 8 weeks, with 4 sessions per week. Pre- and post-intervention assessments included pushups, seated forward bend, standing long jump (SLJ), full-court dribble to layup, 1-minute shooting, 3/4 court sprint (sprint), and a 4-line shuttle run.

**Results:**

The experimental group demonstrated significant improvements in pushups (T = -4.158, *P* < .001), SLJ (T = -10.228, *P* < .001), sprint (T = 2.121, *P* = .047), full-court dribble to layup (Z = -3.921, *P* < .001), and 1-minute shooting (T = -2.373, *P* = .028). The control group showed modest improvements only in the sprint and SLJ. No significant changes were observed in seated forward bend performance in either group.

**Conclusions:**

CF training was more effective than conventional methods in enhancing physical fitness and sport-specific skills among U12 basketball players. These findings support the integration of CF into youth basketball programs to promote athletic development.

## 1. Introduction

The competitive ability of athletes depends on their physical fitness and specific performance, and specific performance was a critical prerequisite for achieving outstanding results in specific sports scenarios.^[1]^ Physical fitness was manifested in muscle strength, endurance, flexibility, etc., which played a crucial role in athletes’ performance during competitions.^[2]^ However, traditional training methods often focused on a single aspect such as training on strength, aerobics, or specific skills, which may be separated from the sports chain during the training process, thereby reducing athletes’ specific performance.^[3]^ With the continuous development of sports training concepts, more and more studies begun to emphasize the importance of comprehensive training in improving athletes’ overall qualities.^[4]^ For young athletes, we must consider their age, gender, and stage of development to ensure the appropriateness and sustainability of the training.

Under this background, CrossFit™ (CF) has emerged as a comprehensive, high-intensity interval training model. CF integrated various elements such as weight training, aerobic training, and physical training, intended to comprehensively improve individuals’ physical fitness and athletic abilities through continuously changing training contents and high-intensity training methods.^[5,6]^ Moreover, the CrossFit Teens™ program was designed specifically to improve the fitness and resistance training skills of teenagers aged between 12 to 18. This program combined 9 core strength exercises in a group training environment.^[7]^ Compared with traditional single training methods, CF emphasizes more on comprehensiveness and can better meet the needs of athletes of different age groups, genders, and developmental characteristics.

CF was appropriate for U12 adolescent basketball players who were at a critical period of physical and mental development.^[8]^ Regular training (RT) methods may not be interesting enough to attract their attention. In contrast, CF diverse forms of movements which stimulated their interest, increased more participation and enhanced the effectiveness of training.^[9]^ It will be easier for adolescent athletes to improve specific skills when they acquire the basic movement patterns and general athletic abilities in their early stages.^[10]^ In addition, adolescence was considered a critical period for developing sports skill abilities, as there were the greatest changes in neuromuscular coordination ability during this period.^[11,12]^ Overall, specialized training is required for the development of young athletes from an early age to maximize their physical attributes and skill improvement. It is crucial for them to achieve their future athletic success by choosing effective training methods to stimulate their interest, increase more participation and enhance the effectiveness of training.

In summary, it’s indispensable to studying on the effect of CF training on U12 adolescent basketball players for improving the training methods, enhancing athletes’ overall qualities, and promoting adolescent athletes’ participation in training. The purposes of this study were as follows: (1) comprehensively evaluating the effects of CF training on the physical fitness and skill levels of U12 adolescent basketball players by comparing CF training methods with RT ones; (2) primarily providing more scientific and effective guidance for the training of adolescent basketball players to lay a solid foundation for their future athletic careers.

## 2. Materials and methods

### 2.1. Participants

This experiment recruited 40 U12 basketball players from basketball training institutions in Yancheng City, Jiangsu Province, China. All participants were male to avoid potential bias due to gender differences. Each participant had received at least 2 years of formal basketball training, and none had any illnesses or sports-related injuries that could affect their performance prior to testing.

This study was conducted in accordance with the ethical guidelines of the Declaration of Helsinki and was approved by the Ethics Committee of Henan University (104754200695). Informed consent was obtained from all participants as well as their parents and/or legal guardians before the commencement of the experiment.

Table 1 shows the basic physical conditions of the basketball players in 2 groups. The independent sample *t* test was conducted on the basic physical conditions of the experimental group (EG) and the control group (CG). It was found that there were no significant differences between the 2 groups (*P* > .05).

**Table 1:** Basic physical characteristics of athletes in the experimental and control groups.

	Experimental group	Control group	T	*P*
Height	158.75 ± 10.622	161.35 ± 7.936	-0.877	.386
Weight	47.40 ± 11.000	50.60 ± 9.075	-1.004	.322

### 2.2. Study design

This study aimed to investigate the effects of 2 different 8-week training interventions on basketball performance variables, including the abilities of jumping and acceleration, shooting skills, and dribbling to layup techniques. All research procedures were completed within the club facilities at the beginning of the preseason (from July–September).

This study adopted a randomized parallel paired design. Players were first matched based on their positions on the team (guards, forwards, and centers) to reduce variability caused by differences in physical demands and skill levels. Within each matched pair, participants were randomly assigned to either the CF training group (CF group, n = 20) or the RT group (n = 20) using a concealed coin toss method conducted by an independent researcher, ensuring allocation concealment. However, assessor blinding was not implemented, which may have introduced potential bias in the performance assessments.

Physical fitness and basketball-specific skill tests were conducted at 3 time points: before the intervention (pretest), during the intervention (mid-test), and after the intervention (posttest). The test items included 30-second pushups (PU), standing long jump (SLJ), seated forward bend (SFB), 1-minute shooting, 3/4 court sprint (sprint), full-court dribble to layup (dribble), and basketball court 4-line shuttle run (shuttle).

During the intervention period, 1 participant in the CF group did not receive the allocated training, and 4 participants withdrew before completing the study. In the RT group, 5 participants were lost to follow-up or discontinued the intervention. As a result, a total of 20 participants in each group completed all assessments and were included in the final analysis.

**Figure 1.**
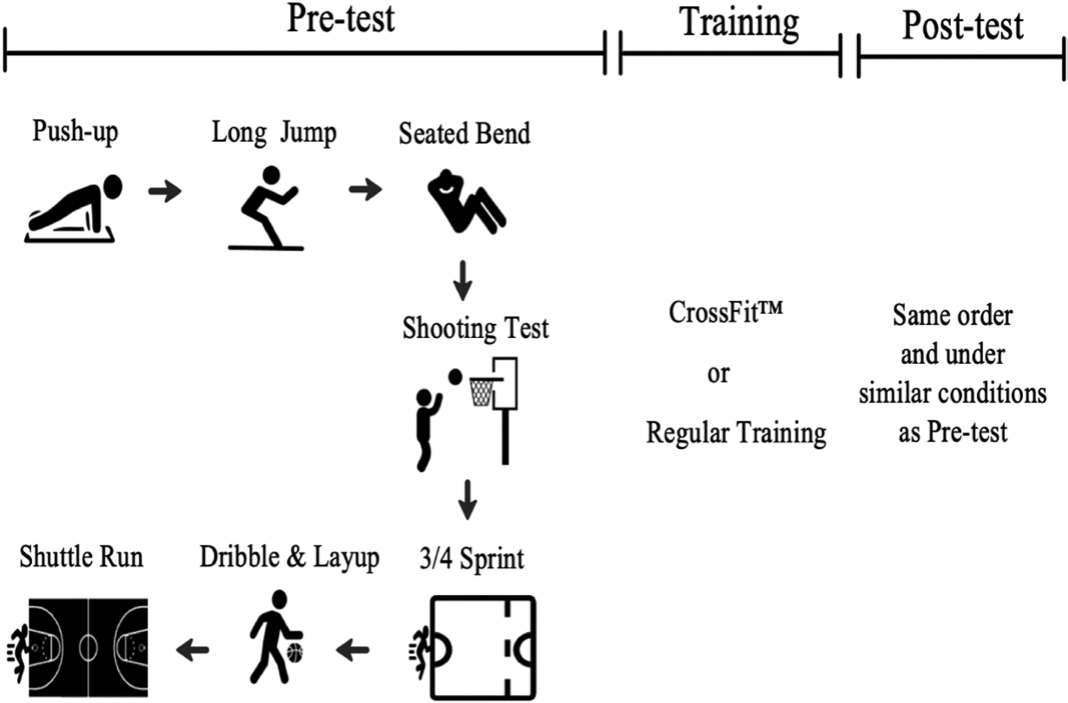
Flowchart of the testing and training procedures, including pretest, training (CrossFit™ or regular training), and post-test.

All participants were familiar with the testing procedures 1 week before the baseline assessment. Additionally, several warm-up sets were recorded before the actual maximal and explosive tests during each testing session to ensure the stability of each measure. Baseline assessments and post-training assessments were respectively made 1 week before the experimental training program and 1 week after that. The tests were conducted in the following orders: PU, SLJ, SFB, 1-minute shooting, sprint, dribble, and shuttle. Prior to testing, all participants had completed warm-ups, including 10 minutes of submaximal running and basketball-specific warm-ups which were sprints, vertical jumps, and joint mobility exercises (Fig. 1). There was a 10-minute rest between tests to minimize the instability or errors caused by fatigue in the subsequent tests.

### 2.3. Graded exercise test

#### 2.3.1. PU test

The PU test is a key indicator for assessing the adolescent players’ upper body strength and endurance that are crucial for the drills such as dribbling, passing, and defense. To be specific, the PU test is appropriate for assessing the muscular endurance level of the adolescent players who are at a critical period of physical development. Thus, the assessment not only contributed to better physical conditions of the players, but also aided the coaches in devising targeted strength training plans to improve their performance.^[13]^

In the test, the participants were required to place their hands on the floor shoulder-width apart with 2 elbows fully extended. Keeping their back and body straight, players lowered themselves until their chests touched the tester’s fist placed beneath the players’ sternums, and then pushed up until the elbows were fully extended. If a participant did not adhere to these specific criteria, the pushups he had done would not be counted. The test was scored based on the number of pushups completed within 30 seconds.

#### 2.3.2. SLJ (cm) test

SLJ serves as an important testing method of the lower body explosive power and jumping ability in the adolescent basketball players. In basketball games, players frequently engage in jumping drills such as rebounding, defense, and scoring. Coaches can know better about the players’ lower body explosive power through the SLJ test so that they will develop more scientifically effective training plans to improve players’ performance.^[14]^ The players were tested with their feet slightly apart and their arms swung during the takeoff phase to support themselves. When they landed correctly and there was no step back, they would take a second test, and the better score in the 2 tests would be retained.

#### 2.3.3. SFB test

In basketball games, flexibility enables players to excite better in the movements of turning, defense, and layup. Flexibility of the adolescent basketball players can be effectively assessed by the SFB test. In the test, the players should be in a seated position with their legs fully extended and arms reaching forward as far as possible. Meanwhile, they slid their hands along a box surface pre-marked with scales, and the length their hands slid would be measured in centimeters (cm). Every participant took the test twice, and the better result would be recorded.^[15]^

#### 2.3.4. One-minute shooting test

**Figure 2.**
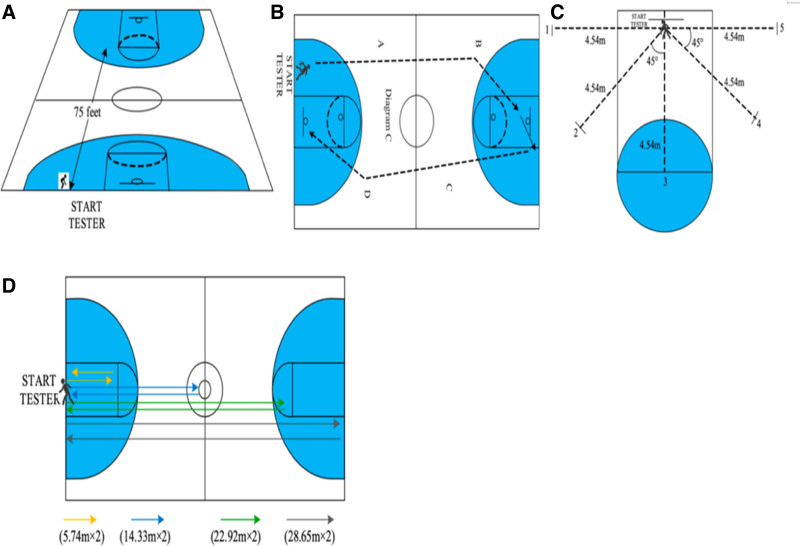
Test protocols used: (A) 3/4 court sprint; (B) full-court dribble to layup; (C) 1-minute shooting; (D) basketball court 4-line shuttle run.

Basketball players’ shooting skills can be quantified by 1-minute shooting test (Fig. 2C) in which every player spends 1-minute shooting from 5 different positions. All these positions are 4.54 m away from the center pole of the basketball hoop, respectively at angles of 0°, 45°, 90°, 135°, and 180°. Upon hearing the whistle, players started from the baseline and attempted to shoot from different directions within 1 minute, immediately rebounding after the shots. Then they shot again, but the shot could not be made from the same direction twice in a row. This test aimed to improve the players’ accuracy and speed in shooting while fostering their capabilities in reaction and rebounding. A successful shot scored 1 point, while unsuccessful shots or shots touching the basketball hoop scored 0 point. The players must stand outside the arc to shoot, and stepping on the arc made the shot invalid. Every participant was tested twice, and the better score was recorded.^[16]^

#### 2.3.5. Sprint test

The sprint test is a crucial assessment method of the adolescent basketball players’ specialized abilities in quick response and starting acceleration (Fig. 2A). Players need to swiftly start and sprint for defense, offense, or rapid transitions in basketball games.^[17]^ In this test, the participants began at the basketball court 2 points behind the baseline. They sprinted as fast as they could towards the opposite free-throw line (75 feet, 22.86 m). The time they spent was measured from the individual’s first movement until they crossed the opposite free-throw line (Smart Speed; Fusion Sport, Brisbane, Australia). Every participant was tested twice, and the better score was recorded.^[18]^

#### 2.3.6. Dribble test

Basketball players frequently dribble to layup for higher scores, which requires strong lower body strength and excellent ball control skills. Dribble is an effective testing method to assess lower body strength and ball control skills of adolescent basketball players (Fig. 2B). The participants were required as follows: before the test, the participants stood at the starting line with the ball; upon hearing the start signal, they dribbled as fast as possible to complete a layup; if the participants missed the goal, they could try again in the same way they dribbled to layup at the first time; after scoring a goal, they picked up the ball and returned to the starting line. The time they spent was recorded from their start-ups to the completion of 2 layups. Every participant took the test twice, and the better score was retained.^[19]^

#### 2.3.7. Shuttle test

During a basketball match, players need to keep running and make frequent transitions between starting and stopping, hence speed endurance and specialized physical fitness are especially important for their performance. The shuttle test is an important assessment indicator for adolescent basketball players’ speed endurance and specialized physical fitness (Fig. 2D). The test rules were as follows: participants started behind the baseline, ran to the free-throw line (5.74 m), and returned to the baseline; then they ran to the half-court line (14.33 m), and returned to the baseline; after that, they ran to the free-throw line on the opposite side (22.92 m), and returned to the baseline. Finally, they completed the full-court run (28.65 m) and got back to the starting position. The total distance covered was 143.28 m. Every participant was tested only once.^[20]^

### 2.4. Training program

Based on the research objectives and the complexity and continuity of the experimental procedures, the training was set to last for 8 weeks, with 4 interventions per week. Before the experiment, a 1-week pre-experiment had been carried out to instruct the participants on the movements and basic requirements of CF training so that they gained a preliminary understanding of the training plan. The training plan of the EG in this study was sourced from the classic daily training plan workout of the day (WOD) recognized by CF officials.^[7]^

Weightlifting, gymnastics, athletics, and other simple functional movements were not only included in the classic WOD of CF training, but also, they were segmented, recombined, and interspersed in the training. After warm-up in each training session, the participants firstly received strength and technique training, and then fulfilled the corresponding classic training tasks on schedule. Every training session was scheduled within 30 minutes, and the principles of intensity, practicality, and variation were embodied in every WOD. The training for the EG was arranged 4 times a week on Mondays, Wednesdays, Fridays, and Sundays. There were 2 cycles with the same training contents in the whole experiment, and each lasted for 4 weeks with a total of 32 training interventions (Table 2). The CG and the EG shared the same training schedules and cycles. The main differences between the 2 groups were the training methods and intervals.

**Table 2:** Training content arrangement for the experimental group.

Week	Section	Content	Sets/time
1.1	WU	20 Russian twists, 20 standing vertical jump, 15 lunges.	3 sets
	WOD AMRAP	20 Sq jumps, 20 tuck jumps, 15 upright push-ups.	20 min
	CD	Standing forward bend, hurdler stretch.	5 min
1.2	WU	12 knee-to-elbow planks, 15 m lateral slides, 20 Sq jumps.	3 sets
	WOD AMRAP	15 pushups, 1-minute squat, 20 box jumps.	20 min
	CD	Seated forward bend, seated side hip stretch	5 min
1.3	WU	15 Sit-ups with bent knees, 10 tuck jumps	3 sets
	WOD AMRAP	1-minute goblet squats, 1-minute cushioned sq jumps, 15 pushups, 2 shuttle runs (15 m each)	3 sets
	CD	Seated side hip stretch, standing single-leg stretch	5 min
1.4	WU	15 Reverse leg curl crunches, 10 butterfly sq jumps, 30-second high knees	3 sets
	WOD AMRAP	15 lunge jumps, 1-minute squat, 15 pushups	20 min
	CD	Hurdler stretch, seated single hip stretch	5 min
2.1	WU	15 box jumps, 30-second plank, 15 sq jumps	3 sets
	WOD AMRAP	16 lateral twisting waist wall pass, 10 overhead squats, 15 box jumps, 50 m sprint	20 min
	CD	Standing quadriceps stretch, supported calf stretch	5 min
2.2	WU	30 standing vertical jumps, 30-second V-sit with resistance, 10 pushups	3 sets
	WOD AMRAP	Tabata intervals, bench-supported extension, high knees, prone back-ups, upright pushups, tuck jumps	2 sets
	CD	Kneeling with unilateral quadriceps stretch, straddling hip stretch	5 min
2.3	WU	30 single under skips/20 double under skips, 30 dead bug exercises, 30 squats	3 sets
	WOD AMRAP	15 basketball thrusters, 50 m sprint, 10 box jumps, 3 shuttle runs (15 m each)	20 min
	CD	Lunge lateral Iliopsoas stretch, supine leg raise with hamstring stretch	5 min
2.4	WU	10 sit-ups with leg raises, 15 basketball thrusters	3 sets
	WOD AMRAP	10 box jumps, 15 upright pushups, 15 half-squat jumps	20 min
	CD	Prone calf stretch, seated forward bend	5 min
3.1	WU	30 sq jumps, 15 reverse leg curl crunches, 30 lunges	3 sets
	WOD AMRAP	10 depth jumps, 15 pushup, 15 lateral waist twisting wall pass, 1-minute hip bridge	20 min
	CD	Supported calf stretch, seated side hip stretch	5 min
3.2	WU	30 sq jumps, 20 Russian twists	3 sets
	WOD AMRAP	1-minute plank, 1-minute left side bridge, 1-minute right side bridge, 1-minute hip bridge, 30 box jumps	20 min
	CD	Dynamic inner thigh stretch, dynamic hip stretch	5 min
3.3	WU	30-s bicycle crunches, 30 hanging leg raises, 30 jumping jacks	3 sets
	WOD AMRAP	15 burpees, 50 m sprint, 20 butterfly sq jumps	20 min
	CD	Half-squat groin stretch, kneeling with unilateral quadriceps stretch	5 min
3.4	WU	20 Russian twists, 30 dead bug exercises, 0-second high knees	3 sets
	WOD AMRAP	15 Lateral Waist Twisting Wall Pass, 1-minute Goblet Squats, 10 Overhead Squats	20 min
	CD	Supported calf stretch, seated side hip stretch	5 min
4.1	WU	30 single under skips/20 double under skips, 20-second high knees, 10 dynamic planks	3 sets
	WOD AMRAP	Tabata intervals, lunge jumps, pushups, tuck jumps, front-to-back jumps, mountain climbers, prone jumping jacks	2 sets
	CD	Straddling hip stretch, standing forward bend	5 min
4.2	WU	30 jumping jacks, 30 dead bug exercises, 10 pushups	3 sets
	WOD AMRAP	40-second hollow body rock, 12 single-leg squats, 15 lunge jumps, 15 m walking lunges	20 min
	CD	Supine quadriceps stretch, single-leg toe touches	5 min
4.3	WU	30 hanging leg raises, 30-second single under skips/20 double under skips, 15 reverse leg curl crunches	3 sets
	WOD AMRAP	8 kipping pull-ups, 10 depth jumps, overhead squats, 1-min cushioned sq jumps	20 min
	CD	Half-squat groin stretch, hurdler stretch	5 min
4.4	WU	10 dynamic planks with abdominal squeeze, 15 jumping jacks, 30 single under skips/20 double under skips	3 sets
	WOD AMRAP	10 depth jumps, 15 pushups, 15 lateral waist twisting wall pass, 15 basketball thrusters	20 min
	CD	Seated forward bend, straddling hip stretch	5 min

In WOD AMRAP, there are no rests or minimal rest intervals between each movement to complete as many rounds as possible within 20 minutes.

This study-controlled training loads by monitoring the heart rates of participants in both groups. Heart rate was measured using the palpation method (primarily at the radial and carotid arteries for basketball players) for 10 seconds immediately after participants completed daily training tasks. The 10-second measurement was then multiplied by 6 to estimate the heart rate per minute. It was essential that the participants’ heart rates in both groups remained within the same range after completing the daily training sessions to ensure that the training loads were comparable between the 2 groups. Prior to the experiment, participants’ resting heart rates in a supine position were recorded in the morning, on an empty stomach, before they woke up. During the experiment, heart rates were recorded the following morning after each training intervention under the same conditions as the baseline measurement. Each recorded heart rate was compared with the baseline to ensure that participants were not overtraining.

### 2.5. Statistical analysis

All statistical analyses were performed using IBM SPSS Statistics for Windows, version 24.0 (IBM Corp., Armonk). Data were collected from pretests and posttests to assess the effects of CF training in the EG and RT in the CG. Descriptive statistics, including mean and standard deviation, were calculated for all variables at each time point.

The normality of data distributions was assessed using the Shapiro–Wilk test, and homogeneity of variances was evaluated using Levene test. For variables that followed a normal distribution and met the assumption of homogeneity of variances (PU, sprints, SFB, and shuttle), paired samples *t* tests were used to compare pretest and post-test results within each group. For variables that did not meet the normality assumption (dribble, SLJ, and 1-minute shooting), the non-parametric Wilcoxon signed-rank test was employed for within-group comparisons. To compare the differences between the EG and the CG, independent samples *t* tests were conducted for analysis.

Effect sizes were calculated using Cohen d to estimate the standardized differences between means. Effect sizes were interpreted as small (d = 0.20), medium (d = 0.50), and large (d ≥ 0.80) according to Cohen guidelines.^[21]^ Statistical significance was set at *P* < .05 for all analyses.

## 3. Research results

### 3.1. The comparative analysis between the EG and CGs before and after the experiment

**Figure 3.**
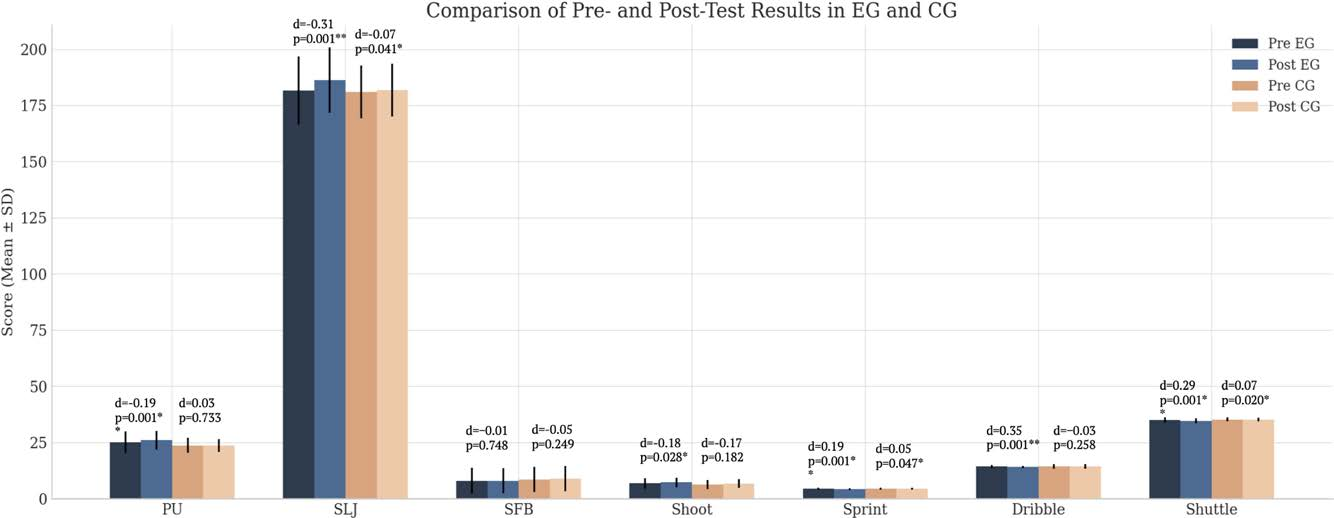
Comparison of pretest and posttest results in the experimental group (EG) and control group (CG). * Significant differences at *P* < .05. ** Highly significant differences at *P* < .01. d = Cohen d; dribble = full-court dribble to layup; PU = pushups; SFB = seated forward bend; shoot = 1-minute shooting; shuttle = 4-line shuttle run; SLJ = standing long jump; sprint = 3/4 court sprint; *p* = *P*-value.

According to Table 3 and Figure 3, in the PU test, the EG improved from a pretest average of 25.25 to 26.15 after training (T = -4.158, *P* < .001, Cohen d = -0.19), while the CG decreased slightly from 23.80 to 23.70 (T = 0.346, *P* = .733, Cohen d = 0.03). In the SFB test, minor changes were observed in both groups: the EG increased from 8.00 cm to 8.05 cm (T = -0.326, *P* = .748, Cohen d = -0.009), and the CG improved from 8.65 cm to 8.95 cm (T = -1.189, *P* = .249, Cohen d = -0.054); however, neither change was statistically significant. For the dribble, the EG improved significantly, with times decreasing from 14.37 seconds to 14.13 seconds (Z = -3.921, *P* < .001, Cohen d = 0.35), while the CG showed no meaningful change (14.39 seconds to 14.42 seconds; T = -1.165, *P* = .258, Cohen d = -0.031).

**Table 3:** Comparison of pretest and posttest scores between experimental group and control group.

Variable	Group	Pretest	Posttest	Growth value	T/Z	*P*-value	Cohen d
Pushups	EG	25.25 ± 4.734	26.15 ± 4.158	0.9	-4.158	.001**	-0.19
	CG	23.80 ± 3.365	23.70 ± 2.812	-0.1	0.346	.733	0.03
Standing long jump	EG	181.60 ± 15.191	186.25 ± 14.581	4.65	-10.228	.001**	-0.306
	CG	181.05 ± 11.736	181.85 ± 11.717	0.8	-2.045	.041*	-0.068
Seated forward bend	EG	8.00 ± 5.777	8.05 ± 5.549	0.05	-0.326	.748	−0.009
	CG	8.65 ± 5.603	8.95 ± 5.753	0.30	-1.189	.249	-0.054
1-min self-shoot and self-rebound	EG	6.85 ± 2.277	7.25 ± 2.124	0.4	-2.373	.028*	-0.176
	CG	6.40 ± 2.037	6.75 ± 1.916	0.35	-1.334	.182	-0.172
3/4 court sprint	EG	4.46 ± 0.417	4.38 ± 0.401	-0.08	9.497	.001**	0.192
	CG	4.47 ± 0.387	4.45 ± 0.378	-0.02	2.121	.047*	0.052
Full-court dribble to layup	EG	14.37 ± 0.686	14.13 ± 0.598	-0.24	-3.921	.001**	0.350
	CG	14.39 ± 0.970	14.42 ± 0.984	0.03	-1.165	.258	−0.031
Basketball court 4-line shuttle runs	EG	35.08 ± 1.142	34.75 ± 1.138	-0.33	12.949	.001**	0.289
	CG	35.37 ± 0.874	35.31 ± 0.838	-0.06	2.532	.020*	0.069

(1) EG stands for the experimental group, and CG stands for the control group. (2) Therefore, paired sample *t* tests were used to compare the differences between pretest and posttest data. For the indicators of full-court dribble to layup, standing long jump, and 1-min shooting, since they do not follow a normal distribution, the Wilcoxon signed-rank test should be chosen to assess the differences in pretest and posttest data. Since the indicators do not follow a normal distribution in the training items of full-court dribble to layup, standing long jump, and 1-minute shooting, the Wilcoxon signed-rank test should be chosen to assess the differences in pretest and posttests data.

* Significant differences at *P* < .05.

** Highly significant differences at *P* < .01.

**Figure 4.**
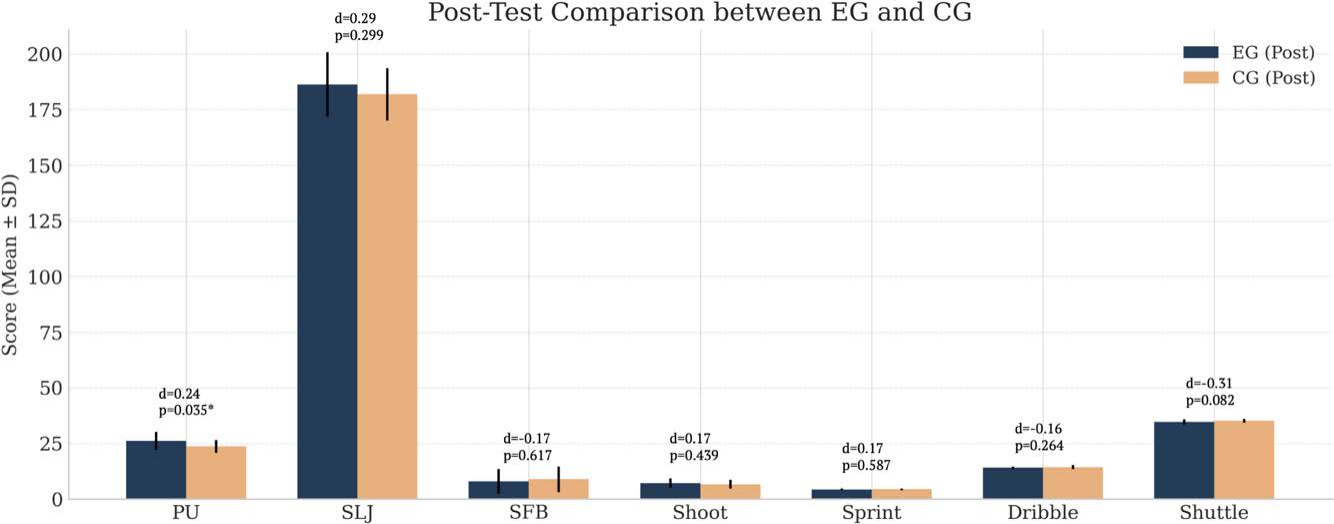
Comparison of pretest and posttest scores (mean ± SD) in the experimental group (EG) and control group (CG). * Significant differences at *P* < .05. d = Cohen d, dribble = full-court dribble to layup, PU = pushups, SFB = seated forward bend, shoot = one-minute shooting, shuttle = 4-line shuttle run, SLJ = standing long jump, sprint = 3/4 court sprint, *p* = *P*-value, SD = standard deviation.

According to Table 4 and Figure 4, independent samples *t* tests were conducted to compare the post-test performance between the EG and the CG. Among all measured variables, a statistically significant difference was found only in the PU test, where the EG outperformed the CG (26.15 ± 4.158 vs. 23.70 ± 2.812; T = 2.183, *P* = .035, Cohen d = 0.244). No significant differences were observed in the other 6 tests, including SLJ (T = 1.052, *P* = .299, d = 0.287), SFB (T = -0.504, *P* = .617, d = -0.166), 1-minute shooting (T = 0.782, *P* = .439, d = 0.174), sprint (T = -0.548, *P* = .587, d = -0.176), dribble (T = -1.134, *P* = .264, d = -0.160), and shuttle (T = -1.785, *P* = .082, d = -0.305). Although some variables showed small to moderate effect sizes, these differences did not reach statistical significance.

**Table 4:** Comparison of experimental and control groups across various performance metrics.

Variable	Experimental group (mean ± SD)	Control group (mean ± SD)	T-value	*P*-value	Cohen d
30-s push-ups	26.15 ± 4.158	23.70 ± 2.812	2.183	.035*	0.244
Standing long jump	186.25 ± 14.581	181.85 ± 11.717	1.052	.299	0.287
Seated forward bend	8.05 ± 5.549	8.95 ± 5.753	-0.504	.617	-0.166
1-min self-shoot and self-rebound	7.25 ± 2.124	6.75 ± 1.916	0.782	.439	0.174
3/4 court sprint	4.38 ± 0.401	4.45 ± 0.378	-0.548	.587	-0.176
Full-court dribble to layup	14.13 ± 0.598	14.42 ± 0.984	-1.134	.264	-0.160
Basketball court 4-line shuttle runs	34.75 ± 1.138	35.31 ± 0.838	-1.785	.082	-0.305

SD = standard deviation.

* Significant differences at *P* < .05.

## 4. Discussion

This study aimed to explore the effectiveness of CF for U12 basketball players on improving their overall athletic qualities by comparing CF training methods with RT ones. The results showed that the CG only enhanced in sprint and SLJ, while the EG achieved significant improvements in pushups, sprint, 1-minute self-shoot and self-rebound (shoot), SLJ, dribble, and shuttle, leading to a positive effect on the physical fitness and skills of U12 youth basketball players.

This study demonstrated that participants receiving CF training achieved significant improvements in increasing pushups within 30 seconds, maximizing the speed in sprint, and enhancing the leap length in SLJ. The results were consistent with the existing findings that CF training effectively enhanced strength, power, and anaerobic capacity.^[22,23]^ However, there were divergences in flexibility between the findings in this study and those in the existing studies. Specifically, no statistically significant differences were observed in either the EG or the CG in the SFB test. In terms of the growth value, the extending length merely increased by 0.05 cm for the EG, while the growth was 0.3 cm for the CG. Neither of the 2 growth was statistically significant, indicating that CF training did not significantly enhance flexibility in this study. This result was contrary to the research findings which proved that CF training significantly improved body composition, flexibility, cardiovascular, and muscle fitness measured in SLJ test.^[7]^

Dribble and 1-minute shooting, are specific tests in basketball training. Dribble primarily tests a player’s speed, ball control abilities, and dribbling stability, while 1-minute shooting evaluates shooting accuracy, in upper and lower body, etc. The EG made noticeable progress in the dribble test with a decrease in time by 0.24 seconds. The decrease is possibly attributed to the movements in CF training which are conducive to enhance the players’ speed and endurance in running. In contrast, the CG’s performance did not improve and the testing time even increased by 0.03 seconds. In the 1-minute shooting test, the EG improved by 0.4 shots, whereas the CG did not show marked enhancement. This may be bound up with the improved endurance and physical fitness of the EG after CF training, leading to more scoring opportunities in games. However, the performance of the CG may have been affected by uneven strength development which resulted in lower shooting accuracy. Overall, CF training is beneficial to improve the dribbling speed and shooting ability of U12 basketball players, which is particularly important for young athletes in the critical stages of physical development and skill acquisition.^[24–26]^

Although there are great advantages of CF training in improving the basketball players’ performance, we have been concerned about potential injury risks associated with the intensity and repeatability of CF training, as well as the technical requirements for certain core exercises. According to data from a minority of participants, CF has led to musculoskeletal injuries and exertional rhabdomyolysis in adults.^[27]^ Moreover, the injury rates in adults are similar to those in reports about weightlifting and gymnastics, and lower than those in competitive contact sports like American football.^[28]^ Despite some injury reports, the injury rate of CF is relatively low compared with that in other competitive contact sports. More importantly, young athletes may benefit from it in the following 3 aspects: firstly, they have more time to train specialized sports skills possibly due to the comprehensive training program and shorter training sessions in CF.^[29]^ Secondly, CF training can help adolescents build confidence. The adolescents may encounter various challenges throughout the training in which they enhance their self-confidence by overcoming the difficulties and making progress, and learning to collaborate with others to achieve common goals. Thirdly, the initial components of CF training include warm-up, preparation, and the Workout of the Day (WOD). Over time, the content of training sessions begins to incorporate more components to better develop specific modalities or technical aspects. Injury rates and severity may vary among different groups of people and in different training environments. Injury rates are contingent upon the factors such as training supervision, program design, and the participants’ experience. Thus, it’s necessary to further investigate these factors in future researches and practices.

As a high-intensity interval training mode CF training has gained much attention in studies on the effects of CF training on aerobic capacity and metabolic responses. Some of them have found that CF training can improve aerobic capacity and metabolic responses. For example, Dobbins et al concluded that long-term CF training significantly improved participants’ aerobic fitness.^[30]^ Similarly, another 2 studies suggested that CF training could effectively improve participants aerobic capacity.^[31,32]^ However, some other studies have indicated that aerobic capacity was not tightly linked to the performance in CF training. Benson et al pointed out that CF training had no direct relation to aerobic capacity, even though it could significantly improve cardiorespiratory functions.^[33]^ Dorgo et al also claimed that short-term CF training did not significantly affect participants’ aerobic capacity.^[34]^ In summary, there remains doubt whether CF training can effectively improve aerobic capacity and metabolic responses. This result reflects the complexity of physiological adaptations to high-intensity interval training modalities, which may be subject to training intensity, duration, frequency, individual differences, etc. Therefore, further studies should focus on identifying the exact relations between CF training and aerobic capacity and metabolic responses, to reveal the potential physiological mechanisms and influencing factors.

This study aimed to assess the effects of an 8-week CF training program on the physical fitness and performance of U12 basketball players, taking into account their physical and psychological developmental characteristics. The findings suggest that CF training may produce short-term improvements in upper- and lower-body strength, dribbling speed, and shooting accuracy. These performance enhancements are particularly relevant for young athletes in critical stages of physical development and motor skill acquisition. Furthermore, the training process demonstrated good feasibility, with high adherence and no major injuries reported, indicating that CF training is acceptable and relatively safe when properly supervised.

However, the results should be interpreted with caution. The relatively small sample size, lack of blinding, and limited intervention period may constrain the generalizability and statistical power of the findings. Additionally, no adjustments were made for multiple comparisons, increasing the risk of Type I errors. While CF training shows potential for integration into youth basketball programs, further longitudinal studies with larger, more diverse populations are needed to verify its long-term efficacy and safety. In future applications, CF protocols should also be tailored to meet the developmental needs of different age groups and athletic levels.

## 5. Strengths and limitations

This study covered multiple physical fitness test items, including PU, sprint, shoot, SLJ, dribble, and shuttle. The inclusion of various test items allowed for a more comprehensive evaluation of the effects of CF training on the overall athletic qualities of U12 basketball players, contributing to more objective and diverse research findings. Additionally, the study adopted a CG design and directly compared CF training methods with RT methods, helping to isolate the effects of CF training by minimizing external confounding factors.

However, there are several limitations to be noted. First, improvements in flexibility, as measured by the SFB test, were not statistically significant in either group. This may be due to the lack of targeted flexibility training components in the training programs, but the specific reasons require further investigation. Second, this study did not control for participants’ baseline physical fitness levels, dietary habits, sleep patterns, or prior training experience (all of which may have influenced the outcomes and reduced the internal validity of the findings). Future research should consider more rigorous control and documentation of these variables to strengthen the credibility of the results. Third, although this study utilized a heart rate monitoring system to provide basic control and recording of training intensity during the intervention, certain limitations remain. Heart rate alone may not fully reflect individual training loads due to its sensitivity to external and internal factors. Future studies should consider incorporating more objective workload indicators such as session RPE, movement repetition counts, or biomechanical assessments (e.g., force plate analysis) to enhance the accuracy and comprehensiveness of training load evaluation. Lastly, while this study highlighted potential risks associated with CF training, it did not delve deeply into safety protocols or risk management strategies. These areas should be further developed in subsequent research to ensure the safe application of CF training, particularly in adolescent populations.

## 6. Practical application

Based on the research findings of this study, it’s recommended that CF training should be integrated into the training regimen for the following considerations: firstly, the diversity and high intensity in CF can meet the demands of the adolescents in physical development and skill acquisition. Meanwhile, this training mode can stimulate the adolescents’ interest and increase their participation in training sessions the combination of CF with traditional basketball training contributes to introducing a comprehensive training program that addresses various aspects including strength, endurance, and flexibility. At the same time, measures should be taken to deepen the integration of CF training into the traditional training. On the one hand, it’s necessary to regularly assess and adjust the training program to ensure that the program is scientific and effective. On the other hand, interdisciplinary collaborations should be encouraged to promote the application of CF in youth basketball training practices for the all-round development of the athletes. These measures will offer young basketball players a more enriching training experience and lay a strong foundation for their future athletic endeavors.

## Author contributions

**Conceptualization:** Jian Gong, Lingqi Li.

**Data curation:** Jian Gong, Lingqi Li.

**Formal analysis:** Jian Gong, Lingqi Li.

**Funding acquisition:** Lingqi Li, Jia Zhang.

**Investigation:** Jian Gong, Lingqi Li.

**Methodology:** Jian Gong.

**Project administration:** Jian Gong.

**Resources:** Jian Gong, Quan Zhou.

**Software:** Quan Zhou.

**Validation:** Jia Zhang.

**Visualization:** Quan Zhou.

**Writing – original draft:** Jian Gong.

**Writing – review & editing:** Jian Gong, Lingqi Li, Jia Zhang.
